# Core microbiomes as a potential fingerprinting method of Western USA dust sources

**DOI:** 10.3389/fmicb.2026.1856083

**Published:** 2026-09-11

**Authors:** DeTiare Leifi, Sarah C. Peterson, Jakob L. Young, Gregory T. Carling, Janice Brahney, Michael C. Duniway, Beth A. Newingham, Nicholas Webb, Travis Nauman, Zachary T. Aanderud

**Affiliations:** 1 Department of Plant and Wildlife Sciences, Brigham Young University, Provo, UT, United States; 2 Department of Biology, Brigham Young University, Provo, UT, United States; 3 Department of Geological Sciences, Brigham Young University, Provo, UT, United States; 4 Department of Watershed Sciences, Utah State University, Logan, UT, United States; 5 US Geological Survey, Southwest Biological Science Center, Moab, UT, United States; 6 Great Basin Rangelands Research Unit, USDA Agricultural Research Service, Reno, NV, United States; 7 USDA Agricultural Research Service Jornada Experimental Range, Las Cruces, NM, United States; 8 Jornada Experimental Range, New Mexico State University, Las Cruces, NM, United States

**Keywords:** aerobiological surveillance, biological pollution, desertification, dust tracking, microbial ecology, mineral dust, wind erosion

## Abstract

**Introduction:**

Changing frequency and intensity of dust emissions impacts ecosystems and human health. Dust carries microbes, nutrients, heavy metals, and other materials that may change environmental biogeochemistry at deposition sites. Identifying dust sources provides key information on where and when mitigation strategies should be employed. However, commonly used geochemical or isotopic tracers are often not capable of distinguishing between geographic regions.

**Methods:**

We explored whether soil bacterial communities may provide distinct fingerprints of dust sources in the western United States. We identified bacterial core communities of dust from ten locations monitored by the National Wind Erosion Research Network (NWERN) with varied land use (cropland, rangeland, and playa), and compared communities to location, soil, and regional characteristics. Samples were collected monthly from Modified Wilson and Cooke (MWAC) samplers, composited by season (spring, summer, and fall), and analyzed using 16S rRNA sequencing.

**Results:**

We found distinct bacterial core communities that reflected dust source characteristics. In order of importance, precipitation levels (*p* = 0.0001), location (*p* = 0.0001), soil texture (*p* = 0.0001), seasonality (*p* = 0.0001), and elevation (p = 0.0002) were correlated with bacterial community composition.

**Discussion:**

Distinct bacterial core communities were associated with site characteristics such as biocrusts, playas, and military base proximity. Our results suggest that the use of core microbiomes may offer a fingerprinting method to identify dust source regions.

## 1 Introduction

Changing frequency and intensity of dust events is a major concern as dust transports nutrients, microbes, heavy metals, and microplastics across vast distances (Moser et al., 2019; Shao et al., 2011; Griffin, 2007, Brahney et al., 2020; Brahney et al., 2024), potentially impacting ecosystems and human health. Dust emission originates from wind-driven erosion of soils in dryland locales (Lee J. A. et al., 2012; Duniway et al., 2019). Dryland areas are sensitive to climate change and land management, which have promoted extensive ecosystem changes to conditions that can be more susceptible to wind erosion (Bestelmeyer et al., 2011; Webb et al., 2025). Understanding dust source locations and the composition of dust can be used to help prioritize restoration, mitigation, and assessing potential health risks (Achakulwisut et al., 2019).

Diverse tracing methods may be employed to understand dust source locations and composition. Geochemical, satellite imagery, and model simulation methods may be employed to trace dust emissions (Ashpole and Washington, 2013; Brahney et al., 2024; Mangum et al., 2024; Yu et al., 2018). Bacterial communities may also be used to trace or fingerprint dust sources, as transported bacteria provide insights into the regional sources of the bioaerosol (Acosta-Martínez et al., 2015). Although geochemical data are more often used in dust fingerprinting due to stability, microbial data provide additional information including relic DNA from source sites that allows for greater specificity, especially when geochemical data alone are insufficient to parse source areas; examination past the phylum level may lead to bacterial community signatures identifying sources of regional dust (Fierer et al., 2012; Lee J. A. et al., 2012). To illustrate, Finn et al. (2021) traced dust loading in Arizona to nearby cotton and livestock farming due to specific bacterial communities (e.g., Micrococcaceae, Acinetobacter, and others) associated with these crops and soils (Johnson et al., 2003; Miethling et al., 2000). Similarly, Dastrup et al. (2018) demonstrated that bacterial taxa including Bacillaceae, Geodermatophilaceae, and Nakamurellaceae are unique to a given airshed, likely reflecting distinct sources from playas and urban areas (Dastrup et al., 2018). Collectively, the aerobiome in terrestrial systems harbors a bacterial signature that may be used for tracing widespread movement like trans-Pacific dust (Smith et al., 2013). Entrained dust harbors thousands of bacterial species in unique combinations, which reflects the ecology, land use history, seasonal signatures, and disturbance regimes of the originating soil (Chen et al., 2021). For example, soils with higher moisture content in the western European plains were dominated by Proteobacteria, but soils in drier conditions contained more Actinobacteria usually from the family Solirubrobacteriaceae (Pershina et al., 2018). Extremophiles and halotolerant bacteria, like Rubrobacteraceae, often dominate in very dry and hyper-saline environments (Connon et al., 2007; Keshri et al., 2013; Pershina et al., 2018). Dust microbial composition also differs across seasons, with higher diversity in spring and summer (March–August) (Ramadoss et al., 2025). Microbial community assembly in dust deposited in arid industrial regions is stochastic, with airborne community composition correlating to air origin and dust particle size (Erkorkmaz et al., 2023; Maloukh et al., 2023; Wang et al., 2025).

To assess the link between dust source region and bacterial community fingerprint, we utilized composited dust samples from Modified Wilson and Cooke (MWAC) samplers at ten National Wind Erosion Research Network (NWERN) sites across the western United States. These sites represent cropland, rangeland, and playa land-use types and a range of soil textures (Table 1). The microbial fingerprints found here are compared with geochemical signatures developed for the same dust samples described in Mangum et al. (2024). We hypothesized that bacterial communities in entrained dust would differ between dust source regions and correlate with seasonal and climate factors. To identify core microbiota, thus characterizing source sites and potentially allowing for emissions tracing, we focused on bacteria that are major and minor constituents shown by relative abundance that are present across many of the samples per site. The use of 16S rRNA microbial analysis to identify dust sources may assist in managing natural and anthropogenically disturbed soil surfaces by identifying specific regions and or land-uses responsible in depositional regions where dust sources are mixed.

**Table 1:** General characteristics of each of the ten National Wind Erosion Research Network (NWERN) sites studied (Webb et al., 2016).

Location	State	Avg rainfall (mm)	Elevation (m)	Soil texture	Dust texture	Land type	Composited samples
Akron	Colorado	421	1,383	Silt Loam	Silt Loam	Cropland	6
CPER	Colorado	320	1,650	Sandy Loam	Sandy Loam	Rangeland	11
El Reno	Oklahoma	815	420	Loam	Silt Loam	Cropland	5
HAFB	New Mexico	278	1,267	Sandy Loam	Silt Loam/Sandy Loam	Rangeland	24
Jornada	New Mexico	250	1,320	Sandy Loam	Sandy Loam/Silt Loam	Rangeland	19
Lordsburg	New Mexico	287	1,267	Sandy Loam	Silt Loam	Playa	3
Mandan	North Dakota	410	591	Silt Loam	Silt Loam	Cropland	3
Moab	Utah	229	1,575	Sandy Loam	Sandy Loam	Rangeland	24
Red Hills	Nevada	200	1725	Loam	Silt Loam	Rangeland	12
Twin Valley	Nevada	200	1,668	Silt Loam	Silt Loam	Rangeland	15

Samples were collected 2018–2020 monthly and composited into spring, summer, and fall seasonal samples. CPER, Central Plains Experimental Range; HAFB, Holloman Air Force Base.

## 2 Materials and methods

### 2.1 Sample collection

**Figure 1:**
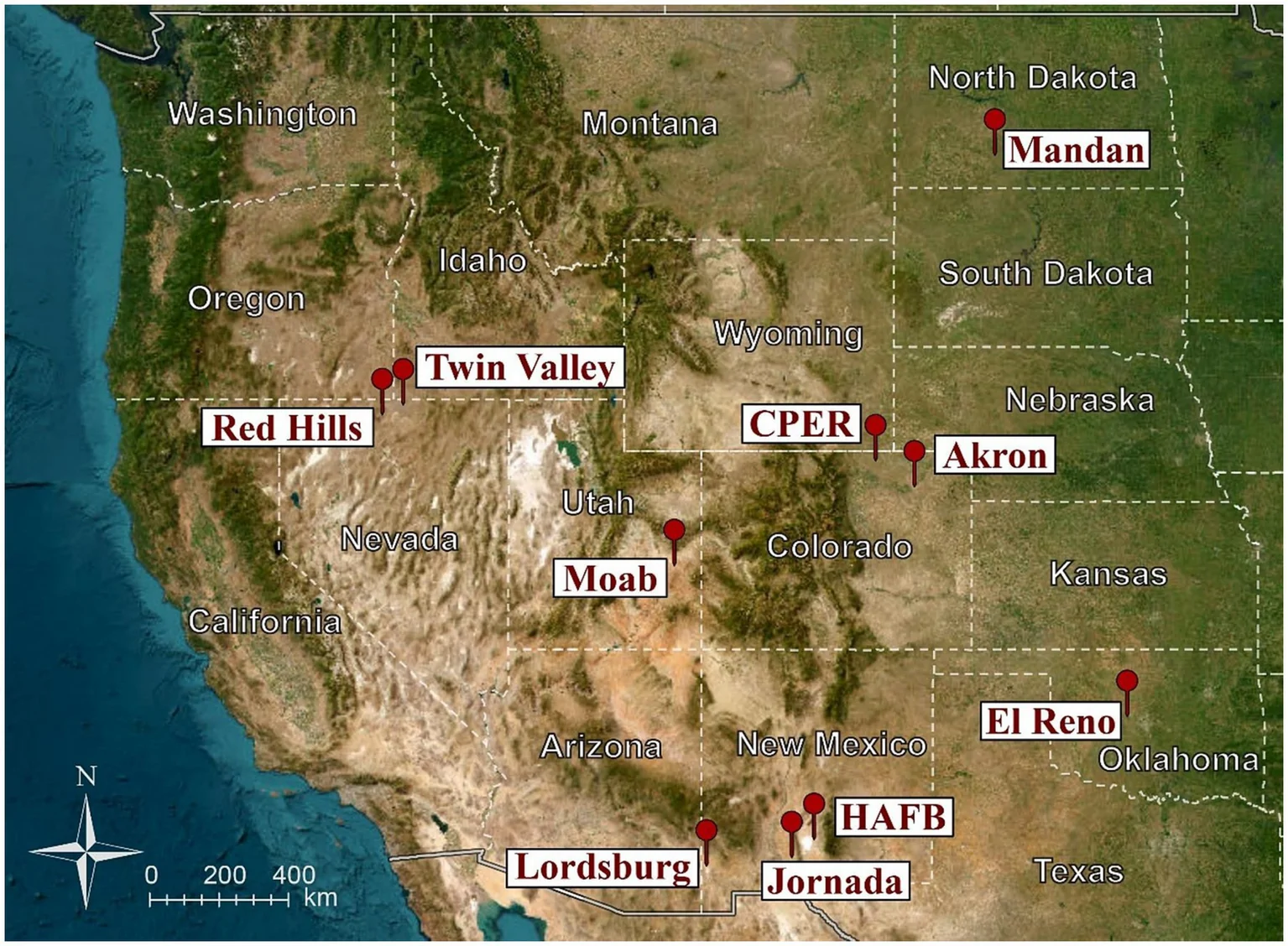
Map of National Wind Erosion Research Network (NWERN) site locations across the Western USA. Map obtained from Mangum et al. (2024). CPER, Central Plains Experimental Range; HAFB, Holloman Air Force Base.

We collaborated with the National Wind Erosion Research Network (NWERN) to analyze dust samples from network sites (Figure 1). NWERN collected aeolian sediment samples monthly using Modified Wilson and Cooke (MWAC) samplers, and samples were later composited into seasons as follows: March–May for spring, June–August for summer, and September–November for fall. MWAC samplers passively collected dust at 10 cm, 25 cm, 50 cm, and 85 cm heights above ground level. Composited samples from the two uppermost heights (50 cm and 85 cm) were used to represent smaller particles (dust) that may travel longer distances relative to larger particles in the lower bins. We selected ten NWERN sites from six states across the western USA with varying land-uses, vegetation, elevation, and soil types (Table 1). These variations were selected for varying microbiota, allowing for correlation between dust source region and bacterial community. The sites included Akron (Colorado), Central Plains Experimental Range (CPER, Colorado), Holloman Air Force Base (HAFB, New Mexico), Jornada Experimental Range (New Mexico), Lordsburg Playa (New Mexico), Moab (Utah), Northern Plains—Mandan (North Dakota), Southern Plains – El Reno (Oklahoma), Red Hills (Nevada), and Twin Valley (Nevada) (Figure 1). Samples were collected from each site according to the collection methods described by Mangum et al. (2024) and Webb et al. (2016). Additional site metadata is publicly available via the NWERN website (Courtright and Webb (n.d.).

### 2.2 Bacterial genomic extraction

16S rDNA gene sequencing analyses were performed to study microbial community composition in composited dust samples from the ten NWERN sites. We extracted genomic DNA from 250 mg sediment samples with the Qiagen DNeasy PowerSoil Pro Kits (Qiagen, Kilden, Germany) with extraction blanks included as negative controls and processed alongside samples through PCR and sequencing. The resulting DNA purity and concentration were measured on a NanoDrop One Microvolume UV–Vis Spectrophotometer with WiFi (Thermo Fisher Scientific, Waltham, MA USA). The extracted DNA was sent to Utah State University Center for Integrated Biosystems for further PCR amplification and Illumina MiSeq (Illumina, San Diego, CA USA) 16S rRNA amplicon sequencing analyses.

### 2.3 Particle size and soil texture analysis

We analyzed the particle size distribution of the composited dust samples using a Sequoia LISST-Portable Particle Size Analyzer Version 3 (Sequoia Scientific Inc., Bellevue, WA USA). We added 20 mL of 3% sodium hexametaphosphate (SHMP) dispersing solution to 100 mg of dust to disaggregate particles in solution overnight. We then placed the bottles on a shaker for 30 min at 200 rpm, then placed the tubes in an ultrasonic bath for 10 min before processing.

Particle size information was used to determine soil texture for each dust sample. Particle size <2 mm was considered clay, between 2 mm and 63 mm was considered silt, and anything >63 mm was considered sand. Particle size fractions of clay and sand were input into TRIANGLE (Gerakis and Baer, 1999) to classify soil texture.

### 2.4 Summary and analysis

Downstream analyses of the 16S rRNA sequences were performed on the bioinformatic pipeline—QIIME 22022.2 (Bolyen et al., 2019). The sequences were demultiplexed and denoised using DADA2 (Callahan et al., 2016). Using amplicon sequence variants (ASVs) that were then aligned with mafft (Katoh and Standley, 2013), the phylogenetic tree was created with fasttree2 (Price et al., 2010). All sequences were rarefied to a sampling depth of 1889 sequences per sample based on the rarefaction curve. Dust samples were analyzed and filtered based on metadata information (McDonald et al., 2012).

The q2-diversity plugin generated alpha-diversity metrics [observed features, Faith’s Phylogenetic Diversity (Faith, 1992)], beta-diversity metrics (weighted UniFrac) (Lozupone et al., 2007), and Bray Curtis distances (Bray and Curtis, 1957). Observed features were used to compare alpha diversity across sites with a Kruskal–Wallis test since ANOVA assumptions could not be met. ASVs were allocated to microbial taxonomies using q2-feature-classifier (Bokulich et al., 2018) classify-sklearn naïve Bayes taxonomy classifier against Silva 138 99% ASVs reference sequences (McDonald et al., 2012).

Each NWERN site and its total bacterial community composition were visualized using a variety of techniques in R (R Core Team, 2026). We utilized the ggplot2 package (Wickham, 2016) to create taxa bar plots that displayed relative abundance of each phylum. Additionally, the microbiome (Lahti and Shetty, 2019), DESeq2 (Love et al., 2014), and phyloseq (McMurdie and Holmes, 2013) R packages were used to identify and summarize abundant and rare bacterial taxa. In QIIME 22022.2 (Bolyen et al., 2019), we performed a principal coordinate analysis (PCoA) using weighted UniFrac distance matrix for each of the individual and grouped NWERN sites. Each site had varying number of samples, feature counts, and total frequencies; therefore, the sampling depth (or rarefaction depth) differed. We then performed a PERMANOVA analysis (Anderson, 2017) with 9,999 permutations using the *adonis2* function to test for the effects of precipitation, soil texture, season, elevation, and location on the microbial community at each site. Some sites (Akron, El Reno, and Mandan) did not have enough samples (*n* < 6) to run a PERMANOVA analysis.

**Figure 2:**
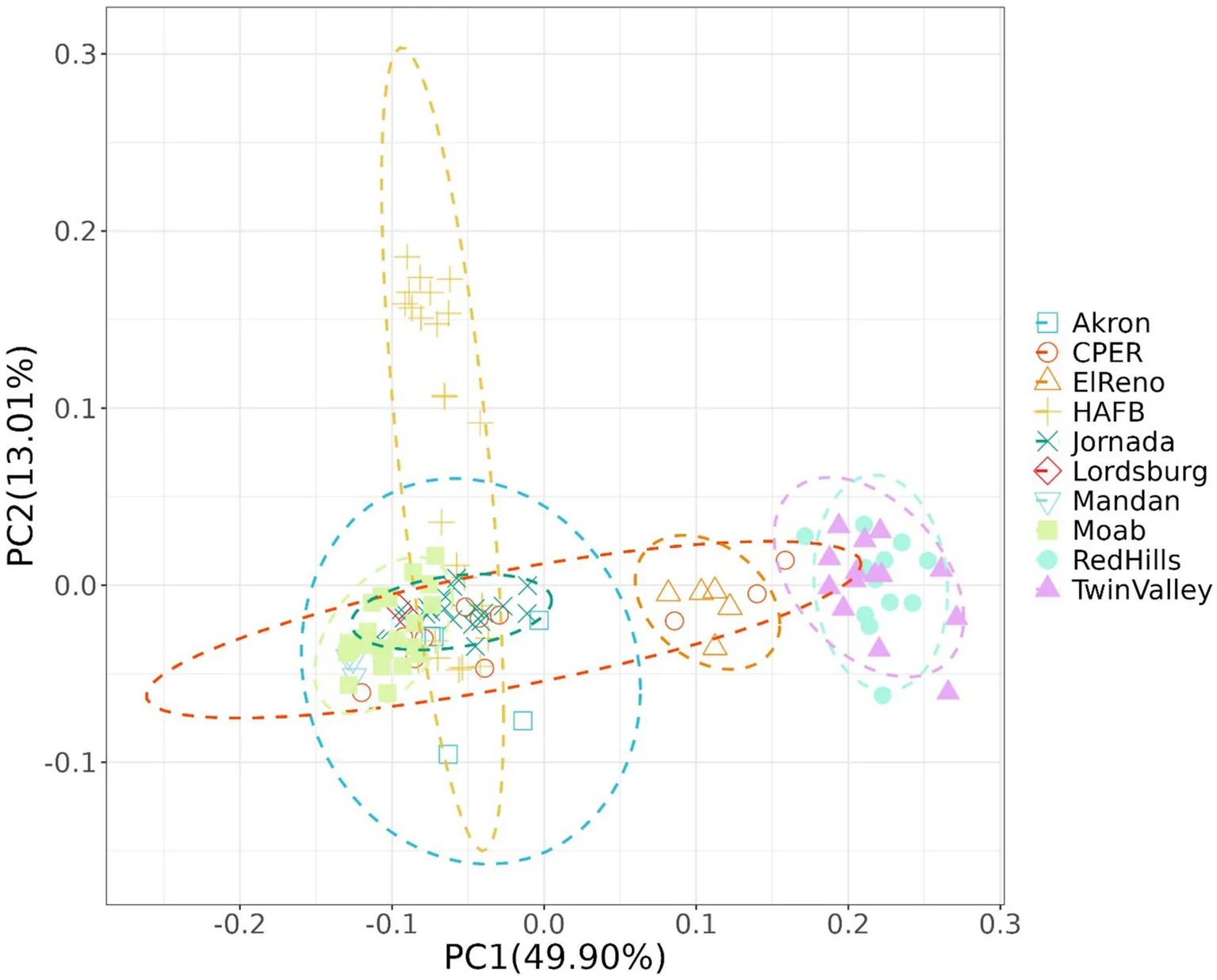
Weighted UniFrac Principal Coordinate Analysis (PCoA) plot of site locations at a 1889 rarefaction threshold. Axis PC1 explains 49.9% of variance among samples, while axis PC2 explains 13.0% of variance. CPER, Central Plains Experimental Range; HAFB, Holloman Air Force Base.

**Figure 3:**
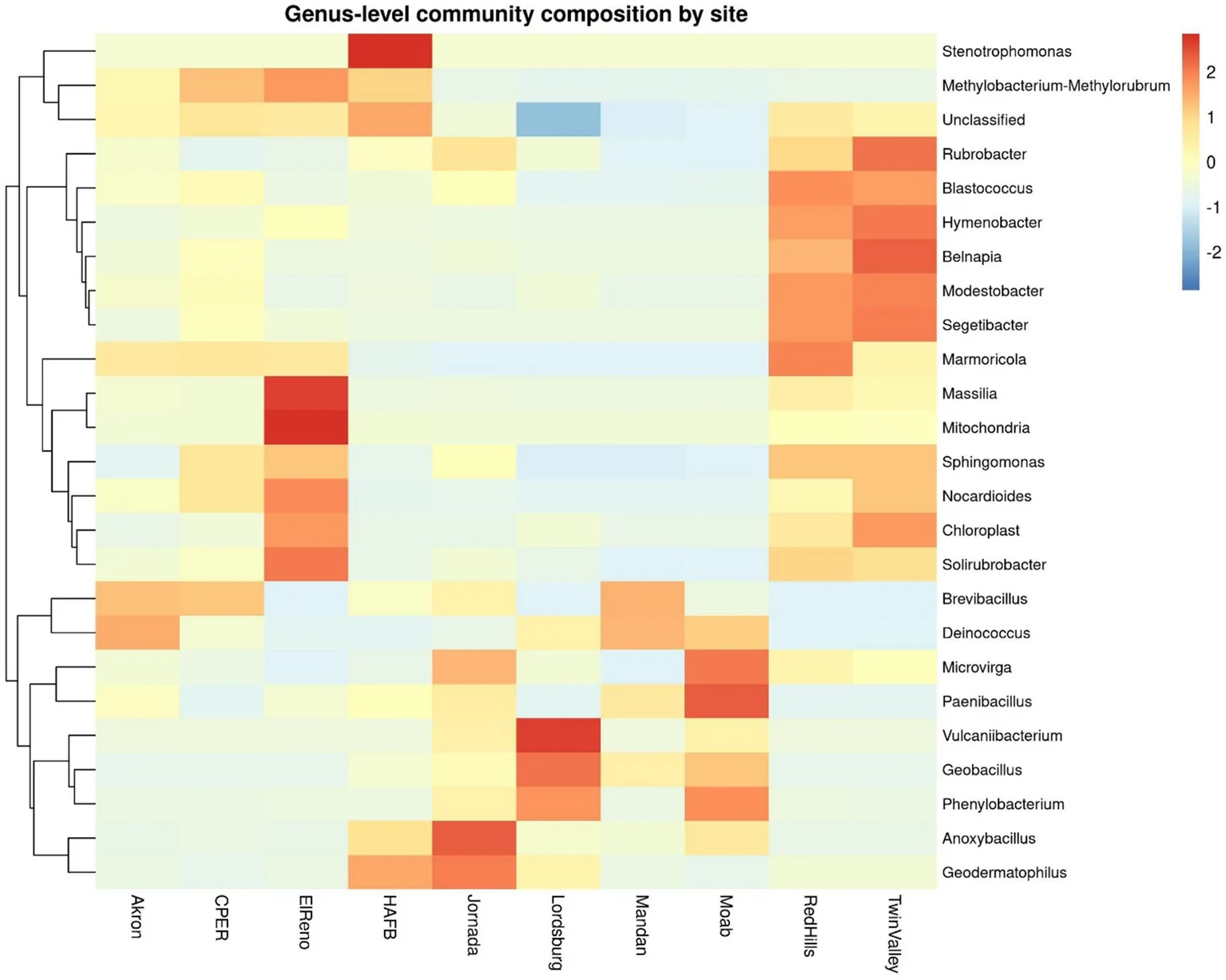
Genus-level heatmap showing patterns in bacterial community composition across National Wind Erosion Research Network (NWERN) sites. Colors represent row-scaled relative abundance of the 25 most abundant genera, with each genus standardized across sites. Columns represent NWERN sampling sites, and rows represent bacterial genera. CPER, Central Plains Experimental Range; HAFB, Holloman Air Force Base.

Core microbiome analyses referenced the weighted UniFrac PCoA plot (Figure 2) and bacterial community taxa bar plots (Figure 3) to determine if NWERN sites shared similar bacteria for grouping. The Lordsburg site was excluded from these analyses because it did not have sufficient samples (*n* = 2). All other sites were analyzed individually. We then assigned ASVs from all the bacterial communities into two abundance classifications: abundant (>1% relative abundance) and rare (<1% relative abundance) (Ogata et al., 2020). These ASVs were present in at least 50% of the samples (50% prevalence) of that site group to be considered in the abundance categories.

We identified bacterial core communities (core) for each site by using Qiime2 filter-features and filter-features-conditionally queries to select for those ASVs that were either abundant or rare. A 50% prevalence cutoff was established to identify microbial taxa that were consistently represented at the sites and therefore categorized common bacterial association at each site. We used taxa bar plots generated by *R. ggplot2* (Wickham, 2016) to visualize the composition of microbial cores that were displayed as percentages of bacterial core taxa. This visualization lists the species that fit the parameters and the proportion of individual species to the total microbial population for that site.

## 3 Results

### 3.1 Unique microbial communities based on location factors

**Figure 4:**
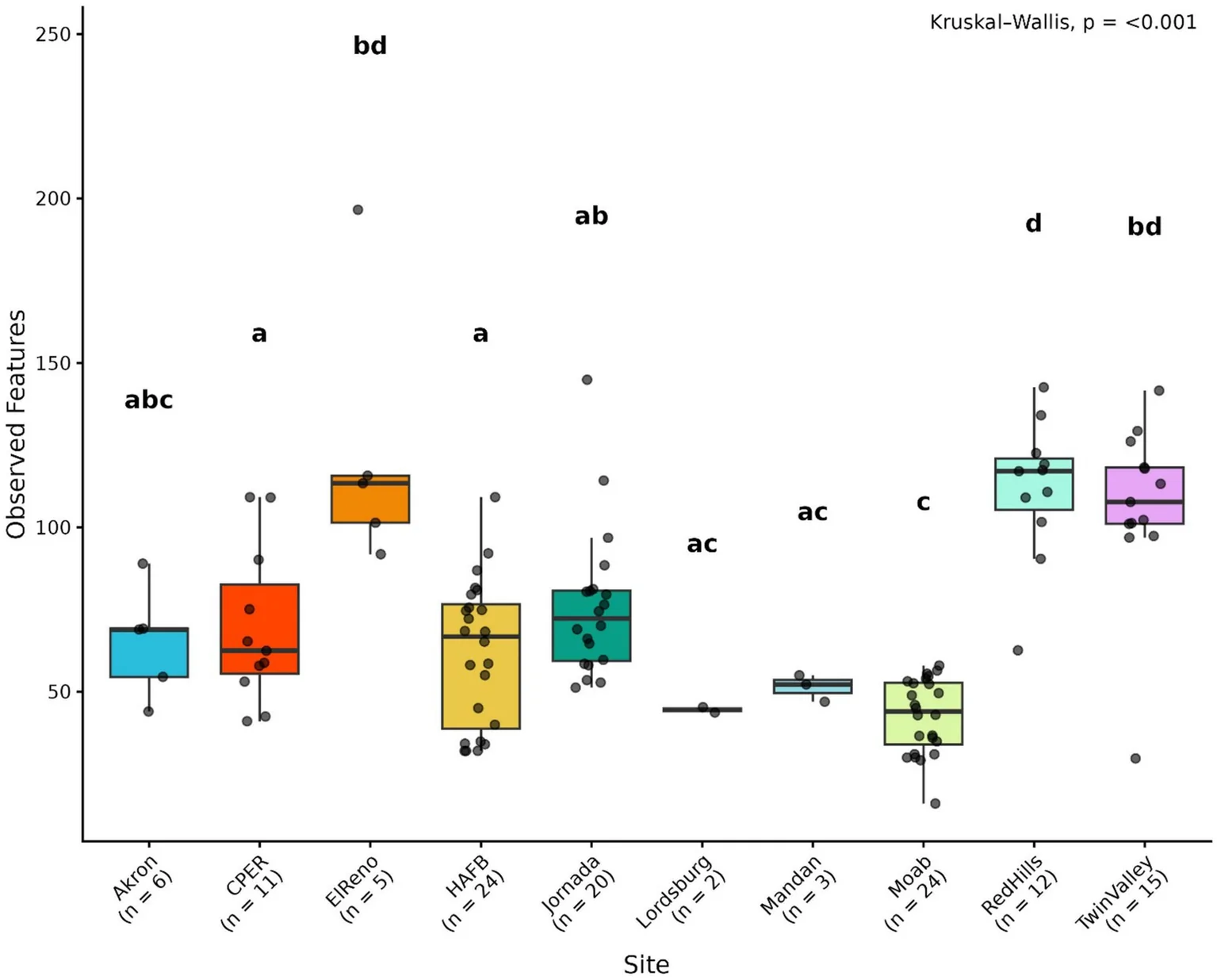
Alpha diversity (observed features) by National Wind Erosion Research Network (NWERN) site location with Dunn letters groupings, *n* per site, and Kruskal–Wallis *p*-value. CPER, Central Plains Experimental Range; HAFB, Holloman Air Force Base.

Samples from some of the NWERN sites plotted distinctly in the PCoA plot, which explained 49.9% of variability along PC1 and 13.0% of variability along PC2 (Figure 2). Precipitation and soil texture predominantly influenced the spread of sites in the PCoA plot, as suggested by the PERMANOVA results (BH-adjusted *p* = 0.0001, 0.0001 and *R*^2^ = 0.25, 0.12, respectively). The Red Hills and Twin Valley sites clustered together along PC1 and PC2. Generally, Red Hills and Twin Valley sites were alike, while all others were individually distinct. El Reno showed distinct clustering on PC1, while Moab, Mandan, Akron, Jornada, and CPER cluster roughly together along both axes. HAFB showed a distinct cluster primarily on PC2, though some samples clustered with other sites. Weighted Unifrac PERMANOVA results found precipitation, location, soil texture, season, and elevation to be significant predictors of microbial communities (Table 2); Bray–Curtis PERMANOVA results found the same five factors to be significant predictors (BH-adjusted *p* < 0.05). A heatmap at the genus level demonstrated distinct bacterial communities in each location (Figure 3). Alpha diversity differed significantly among sites for observed features (Kruskal–Wallis, *p* < 0.0001). Red Hills, Twin Valley, and El Reno showed the highest observed features (Figure 4) and the highest Faith’s phylogenetic diversity with 9.05 ± 1.75, 9.58 ± 2.46, and 11.0 ± 1.48, respectively. Extraction blanks yielded no detectable sequence reads.

**Table 2:** PERMANOVA of weighted UniFrac distance of National Wind Erosion Research Network (NWERN) sites, with *p*-value, *r*-squared value, and degrees of freedom (df) for each variable in the model (precipitation, location, soil texture, season, and elevation).

PERMANOVA—NWERN sites				
Variable	*p*-value	*p*-adj	*r* ^2^	df
Precipitation	0.0001*	0.0001*	0.248	2
Location	0.0001*	0.0001*	0.229	5
Soil Texture	0.0001*	0.0001*	0.12	3
Season	0.0001*	0.0001*	0.064	2
Elevation	0.0002*	0.0002*	0.033	2

*represents *p* < 0.05.

### 3.2 Abundant and rare core microbiomes

Several taxa were shared across multiple sites or endemic to a single site in the abundant and rare cores (Tables 3, 4). Among the abundant core, six Firmicutes members and one Proteobacteria were shared across multiple sites. Abundant bacteria endemic to a single site included additional phyla Actinobacteriota, Cyanobacteria, Deinococcota, and Myxococcota. In the rare core, four Firmicutes members, one Proteobacteria, and one Actinobacteriota were shared across multiple sites. Bacteroidota was endemic in the rare core of El Reno, Jornada, Red Hills, and Twin Valley.

**Table 3:** Abundant bacteria shared across multiple sites (‘Shared’) and endemic to a single site (‘Endemic’), color-coded by phylum. Red Hills and Twin Valley sites share a column (“RH + TV”) due to the lack of difference between their abundant cores.

Abundant bacteria (family level)		Akron	CPER	El Reno	HAFB	Jornada	Moab	Mandan	RH + TV
SHARED	Bacillaceae	✓	✓		✓	✓			
	Brevibacillaceae	✓	✓		✓	✓		✓	
	Bacillaceae *Bacillus*		✓	✓					
	Bacillaceae *Anoxybacillus*				✓	✓	✓		
	Bacillaceae *Geobacillus*				✓	✓	✓	✓	
	Symbiobacteraceae				✓	✓			
	Beijerinchkiaceae					✓	✓		
ENDEMIC	Peptostreptococcaceae	✓							
	Solirubrobacteraceae			✓					
	Chloroplast			✓					
	Planococcaceae			✓					
	Myxococcaceae			✓					
	Mitochondria			✓					
	Oxalobacteraceae			✓					
	Sphingomonadaceae				✓				
	Xanthomonadaceae *Stenotrophomonas*				✓				
	Geodermatophilaceae					✓			
	Xanthomonadaceae					✓			
	Paenibacillaceae *Paenibacillus*						✓		
	Caulobacteraceae						✓		
	Deinococcaceae							✓	
	Paenibacillaceae *Cohnella*							✓	
	Paenibacillaceae *Paenibacillus thermoaerophilus*							✓	
	Paenibacillaceae (uncultured)							✓	
	Geodematophilaceae *Blastococcus*								✓
	Geodematophilaceae *Modestobacter*								✓
	Frankiales (uncultured)								✓
	Micrococcaceae								✓

CPER, Central Plains Experimental Range; HAFB, Holloman Air Force Base.

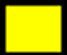
 Actinobacteriota; 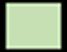 Cyanobacteria; 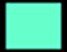 Deinococcota; 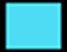 Firmicutes; 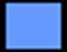 Myxococcota; 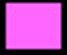 Proteobacteria.

**Table 4:** Rare bacteria shared across multiple sites (‘Shared’) and endemic to a single site (‘Endemic’), color-coded by phylum.

Rare bacteria (family level)		Akron	CPER	El Reno	HAFB	Jornada	Moab	Mandan	RH + TV
SHARED	Bacillaceae	✓	✓		✓		✓		
	Brevibacillaceae	✓	✓			✓	✓	✓	
	Bacillaceae *Anoxybacillus*				✓	✓			
	Bacillaceae *Geobacillus*				✓	✓			
	Beijerinckiaceae		✓		✓				
	Micrococcaceae			✓					✓
ENDEMIC	Erysipelotrichaceae	✓							
	Clostridiaceae	✓							
	Sphingobacteriaceae			✓					
	Planococcaceae			✓					
	Oxalobacteraceae *Massilia*			✓					
	Nocardiaceae				✓				
	Xanthobacteraceae				✓				
	Xanthobacteraceae *Bradrhizobium*				✓				
	Xanthomonadaceae				✓				
	Chitinophagaceae					✓			
	Symbiobacteraceae					✓			
	Geodematophilaceae								✓
	Chitinophagaceae *Segetibacter*								✓
	Sphingomonadaceae								✓
	Oxalobacteraceae *Noviherbasprillum*								✓

Red Hills and Twin Valley sites share a column (“RH + TV”) due to the lack of difference between their rare cores. CPER, Central Plains Experimental Range; HAFB, Holloman Air Force Base.

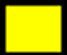
 Actinobacteriota; 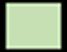 Bacteroidota; 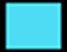 Firmicutes; 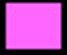 Proteobacteria.

### 3.3 Seasonal changes in microbial communities

Season (March–May for spring, June–August for summer, or September–November for fall) was a significant predictor of bacterial community composition (Table 2). Seasonal shifts in the bacterial community were observed in CPER, HAFB, Jornada, Red Hills and Twin Valley samples. There was no seasonal change in microbial population in the Moab samples.

PERMANOVA analysis indicated that at CPER, season (BH-adjusted *p* = 0.014) and year (BH-adjusted *p* = 0.003) were factors influencing the total microbial community. At HAFB, only season contributed to the bacterial composition (BH-adjusted *p* = 0.003). Similar to HAFB, season at the Jornada site was also a factor influencing bacterial species distribution (BH-adjusted *p* = 0.014). Unlike other sites, season did not influence the bacterial composition at Moab (BH-adjusted *p* = 0.11). Bacterial communities at Red Hills and Twin Valley were primarily driven by season (BH-adjusted *p* = 0.003) and secondarily by year (BH-adjusted *p* = 0.053).

## 4 Discussion

### 4.1 Variable microbial communities between dust source regions

In this study, we aimed to determine whether dust microbial data, including abundant and rare cores, taxa present, and relative abundances, could be used to fingerprint dust source regions. We found distinct communities associated with key land uses, landscape characteristics, and seasons. Similar land uses and site characteristics yielded strong microbial similarities, highlighting that dust microbial data may be used to evaluate contributing source regions, though the current work is descriptive rather than predictive. For example, Red Hills and Twin Valley are both located in Nevada and have similar elevation, precipitation levels, soil texture, land-use management, and suffered burns from the Martin Fire in 2018. Red Hills and Twin Valley were distinct because they contained relatively high abundances of Actinobacteria, Proteobacteria and Bacteroidota. These bacteria may be indicators of this geographic region type, i.e., low-precipitation, high-elevation deserts. Actinobacteria are often found in hyper-arid soils like the Atacama Desert (Azua-Bustos et al., 2015; Crits-Christoph et al., 2013; Drees et al., 2006) and possess a negative correlation to average soil relative humidity (Neilson et al., 2017). Further, Red Hills and Twin Valley had the lowest relative abundances of Firmicutes at 4.3 ± 2% and 2.3 ± 2%, respectively while other sites had higher relative abundances of this phyla ranging from 19.8–87.2%. Firmicutes produce endospores and are commonly found in highly degraded soils as these taxa may withstand harsh climates and environments (Filippidou et al., 2016). Differences between the Red Hills and Twin Valley sites compared to other studied sites may also be due to both sites experiencing wildfire disturbance in 2018; wildfire has been shown to decrease microbial carbon, soil nutrient levels, and enzyme activity (Vourlitis et al., 2022). In contrast, the Jornada and HAFB sites also represent relatively near sites with similar land-use types, precipitation, and elevation but showed somewhat distinct microbial signatures. Though the two sites shared abundant and rare core bacteria, the HAFB total microbial community clustered separately in PCoA analysis. The two sites share similar precipitation levels but differ primarily in soil type, with HAFB dominated by gypsum and biocrusts. Gypsum application to soils has been shown to alter microbial activity and organic carbon mineralization (Liu et al., 2025), which may explain the distinct clustering shown by HAFB samples—with the HAFB site being directly downwind of the White Sands gypsum dunefield. These examples illustrate the impacts of elevation, precipitation, disturbance, and soil type on aeolian sediment microbial communities.

### 4.2 Abundant core and rare core in relation to climate and land-use type

Site core microbiomes (abundant versus rare) demonstrated that within an area, some bacteria were generalists while others were specialists based on the local climate and characteristics. Understanding microbial similarities and differences can be used for developing fingerprints for dust source regions in the western USA. The Akron abundant core shared two common species with CPER from family Bacilliaceae and Brevibacilliaceae. As Firmicutes members, these taxa are gram positive bacteria and can form endospores to adapt against atmospheric stressors like UV radiation and desiccation (Maki et al., 2019; Nicholson et al., 2000), which allows them to dominate dust samples (Kakikawa et al., 2008; Maki et al., 2008; Xu et al., 2019). The CPER site does not have any unique bacteria in either its abundant or rare core. The two bacteria shared with only one other site are *Bacillus* and *Methylobacterium-Methylorubrum,* which are resistant to radiation and desiccation and are plant-associated (Innerebner et al., 2011; Knief et al., 2010; Cai et al., 2022). The El Reno site contained endemic bacteria in both its abundant and rare core: genera *Solirubrobacter* and *Massilia*, which are associated with arid regions and ecological adaptability, respectively (Jiang et al., 2021; Amirhosseini et al., 2025). The HAFB site shared abundant core bacteria (*Anoxybacillus*, *Geobacillus*, *Brevibacillus*, Symbiobacteraceae) and rare core bacteria (*Anoxybacillus*, *Geobacillus*, *Methylobacterium*-*Methylorubrum*) with other sites; these taxa are common in soils, water bodies, and biocrusts, which explains their shared presence across multiple sites (Zeigler, 2014; Sheu et al., 2015). The Jornada site had the same Firmicutes members as the HAFB location; these genera (*Anoxybacillus*, *Geobacillus*, *Brevibacillus*) and families (Bacillaceae and Symbiobacteriaceae) are generalist species with high relative abundance across multiple sites and may be descriptive of the general New Mexico area. *Anoxybacillus* and *Geobacillus* were members of the abundant Moab core and were shared with Jornada and HAFB. The abundant core in Red Hill and Twin Valley contained only Actinobacteria species, which are common in hyper-arid soils (Neilson et al., 2017) and not found in abundant cores from the other sites. In the rare core of Red Hills and Twin Valley, genus *Noviherbaspirillum*, a Proteobacteria, and *Segetibacter uncultured_bacterium* in the Chitinophagaceae family of Bacteroidota were unique bacteria to these sites; these taxa are found in grassland soils (Chaudhary and Kim, 2017) and are associated with smaller aeolian particle sizes, respectively (Polymenakou et al., 2008). Prior to the Martin Fire, Red Hills and Twin Valley were sagebrush-dominated; grassland-associated microbiota may be linked to shifts following disturbance. These findings are limited by uneven samples sizes between sites, rarefaction depth, and exclusion of sites without enough data; however, the use of abundant and rare core microbiomes to characterize sites demonstrated valuable trends.

### 4.3 Seasonal change in microbial community

Seasonal shifts, especially in sites with large seasonal differences in precipitation, correlated with changes in microbial communities. Changes in temperature, precipitation, and humidity may influence soil bacterial communities, as some bacterial taxa may be more sensitive to changes in humidity, precipitation, and temperature (Cai et al., 2022). There was no seasonal influence on bacterial community composition at the Moab site; recent severe drought may have limited seasonal variability, likewise affecting microbial community structure (Ochoa-Hueso et al., 2018). At the CPER site, 70% of the annual precipitation occurs during spring and summer, which may explain the shift in bacterial distribution across seasons at this site, especially given demonstrated shifts in arid soil microbiota following rainfall (Demergasso et al., 2023). At the HAFB and Jornada sites, 50 and 60% of annual precipitation occur during the months of June to September, respectively. Red Hills and Twin Valley receive most of their precipitation as winter snow, which may be another contributing factor for the difference in bacterial community composition at these sites relative to the other study sites. Though winter samples were not available for this study, sediment flux is typically lower and has shorter-range transport capabilities in winter months (Treminio et al., 2025). The observed significant seasonal variations in bacterial communities at nine of the ten sites may be linked to differences in precipitation patterns and air temperature (Xiao et al., 2023). These seasonal findings are somewhat limited since it is not clear to what extent each factor operates independently versus interactively. Coupled with site characteristics such as soil type and precipitation levels, seasonal shifts explain key microbial community differences.

### 4.4 Microbial and geochemical fingerprinting

Our finding of site-specific differences points to the ability to generate unique fingerprints using microbial communities. This newly emerging method provides promise for dust tracing studies upon further analysis and with additional descriptive methodology. Although the fingerprinting method has been implemented for suggested use in forensic casework (Foster et al., 2023), there may be new applications in ecology, especially in regions impacted by high dust deposition. Microbial data mirrored geochemical signatures from the same samples, which showed distinct characteristics for HAFB and strong similarities between Red Hills and Twin Valley (Mangum et al., 2024). The findings differed primarily with regards to the Lordsburg site, which showed unique geochemical characteristics but shared similarities with other sites microbially (Mangum et al., 2024). This result was unexpected given that Lordsburg is a playa site and may indicate that bacteria do not differentiate between playas and other land-use types as distinctively as geochemical data, which show unique signatures for playas (Carling et al., 2020). This difference highlights the importance of combined microbial and geochemical data for use in dust fingerprinting. Recipient ecosystems of dust deposition may exhibit increased sensitivity to dust events depending on pre-existing conditions, and lacustrine recipient sites have shown differences in primary production and phyto- and bacterio-plankton community composition (Wen et al., 2025; González-Olalla and Brahney, 2025). These differences may be anticipated and studied with more well-developed characterization of dust source locations by microbial fingerprinting. Further work fingerprinting dust sources, including dust emission sites in different regions, by microbial signature and geochemical data combined will allow for tracing of dust deposition path from source to sink.

## Data Availability

The datasets analyzed for this study can be found in the NCBI Sequence Read Archive at http://www.ncbi.nlm.nih.gov/bioproject/1453572.
