# Supplementary material for: Core microbiomes as a potential fingerprinting method of Western USA dust sources
Source: Front Microbiol. 2026 Sep 11;17:1856083. doi: 10.3389/fmicb.2026.1856083 (PMC13612543; doi:10.3389/fmicb.2026.1856083)
Supplement: Supplementary file 1 [file Table_1.docx]

Supplementary Material

# 1 Supplementary Data

Site Descriptions

**Akron**

The Akron location is managed as a cropland agricultural site with no-till in the winter and summer fallow rotation of wheat, corn, and millet. It is located approximately 7km east of Akron in Washington County, Colorado and 154km southeast of the CPER site. The Akron site had an elevation of 1383m above sea level and the terrain is characterized by level plains and rolling hills. The annual average precipitation level was 421mm where 72% of the annual rainfall typically occurred between the months of April and August. In July, the average monthly minimum and maximum temperatures ranged between 15° C and 31° C and in January, the temperature ranged from -9° C to 3.5° C. The surface soil texture was a loam to Silt Loam. The slope was 0% at the site, whereas the nearby terrain slopes ranged from 0 to 3%.

**Central Plains Experimental Range (CPER)**

The CPER site was a rangeland site that was grazed by cattle. This site is encompassed by the Pawnee National Grassland in the north and located 40km northeast of Greeley, Colorado. The elevation of this site was 1650m above sea level and the area was characterized by rolling hills and terraces. The average annual rainfall is 320mm where 70% of the annual precipitation occurred between the months of April and September. In July, the average monthly minimum and maximum temperatures ranged between 14° C and 34° C and in January, the temperature ranged from -9° C to 8° C. The vegetation at this site was predominantly blue grama and a mixture of other warm season grass types. In areas not impacted by grazing cattle or animals biocrusts were dominantly present. The surface soil texture was a sandy clay loam above fine sandy loam. This site had a slope of 0% however the surrounded terrain slopes range from 0 to 15%.

**Holloman Air Force Base (HAFB)**

The HAFB site was a protected military air force base located in the northern Chihuahuan Desert and managed as a rangeland and were grazed by cattle and gemsbok. This site was in south-central New Mexico within the Tularosa Basin and approximately 67km northeast of the Jornada site. To the west of HAFB was the San Andres Mountains and to the east the Sacramento Mountains. Immediately to the west of the site lay the White Sands National Monument and White Sands dunes. The elevation of this site was at 1267m above sea level with some nearby summits in the Lincoln National Forest that reached 3652m. The average annual rainfall was 278mm where 50% of the annual precipitation occurred between the months of June and September. The average annual maximum and minimum temperature for this site ranged between 25° C and 8.5° C. The vegetation at this site was a mixture of native grasses, forbs, and shrubs. The soil surface consisted of biocrusts that were visibly present as darkened crusts. Species of lichen were also attached to biocrusts at the soil surface. The soil texture was fine sandy loams, while subsurface soil texture was coarser sandy loams. This site has a slope of 0%.

**Jornada Experimental Range (Jornada)**

The Jornada Experimental Range was a part of the USDA-ARS Long Term Agro-ecosystem Research (LTAR) network. This location was a rangeland site that was grazed by cattle and located in south-central New Mexico within the Jornada Basin. Like the HAFB site, the Jornada site was in the northern Chihuahuan Desert, however it was bordered by the Rio Grande Valley to the west and the San Andres Mountains to the east. The elevation of this site was 1320m above sea level with nearby summits of the San Andres Mountains reaching 2747m. The average annual rainfall for the Jornada Basin was 250mm where 60% of the annual precipitation occurred between the months of June and September. The average annual maximum and minimum temperature for this site ranged between 25° C and 5° C. The vegetation at the Jornada site consisted of a mixture of native species of grasses, perennial forbs, and shrubs. The surface soil consisted of weak biocrust presence. The soil texture was sandy loam with subsurface soil textures ranging from sandy loam to sandy clay loam. The slope at this site was less than 0.5%.

**Lordsburg Playa (Lordsburg)**

The Lordsburg Playa site was situated on a dried lakebed and managed as a rangeland site that was grazed by cattle. This site was located approximately 20km southwest of Lordsburg, New Mexico. It was in the northern Chihuahuan Desert with the Peloncillo Mountain range to the west and the Pyramid Mountains to the east. This site was at an elevation of 1267m above sea level. There was little to no vegetation at this location.

**Moab**

The Moab site was in south-eastern Utah on Bartlett Flat and categorized as a rangeland that was grazed by native herbivores and cattle. To the west of this site was the Green River and Labyrinth Canyon and to south was the Canyonlands National Park – Island in the Sky District. This site had an elevation of 1575m above sea level and was situated on a mesa or flat-top mountain. The average annual rainfall in this area was approximately 229mm. Precipitation fell consistently throughout the years, with slightly more rainfall occurring in October and slightly less occurring in June, when compared to other months. In July, the average monthly minimum and maximum temperatures ranged between 19° C and 33° C and in January, the temperature ranged from -6° C to 3° C. The vegetation type at this site was predominantly warm season grasses with some woody species. The soil texture was loamy fine sands with very fine sandy loams at the subsurface level. The slope at this site was less than 1%.

**Northern Plains - Mandan**

The Mandan site was a part of the Long Term Agro-ecosystem Research (LTAR) network and managed by the USDA Northern Great Plains Research Laboratory (NGPRL). The Mandan site was categorized as a dryland cropping area under no-till management with various cropland studies and treatments. The main crops that were grown at the Mandan site were sunflowers, soybean, and canola. This site was about 6km south of Mandan, was within the Temperate Steppe region of North Dakota and situated on the Missouri Plateau. The elevation level for this site was 593m above sea level. The mean annual rainfall was 410mm at the site with approximately 330mm of rainfall that occurred the growing seasons of April to September. The daily mean temperature in the summer was 21° C and -11° C in the winter, with an annual average temperature of 4° C. The site topography consisted of rolling hills (0 – 3% slope). The soil texture at the site was fine silt loam.

**Southern Plains – El Reno**

The El Reno site was a no-till winter wheat agricultural site. It was in El Reno, Oklahoma and about 50km west of Oklahoma City. It was managed by the USDA Agriculture Research Service Grazinglands Research Laboratory and was a part of the Long Term Agro-ecosystem Research (LTAR) network. The site elevation was 420m above sea level. The average annual rainfall was 815mm where 70% of the annual precipitation occurred in spring and summer. The mean temperature at the site ranged from 0.5° C in January to 30° C in August. The dominant vegetation at the site was winter wheat with annual grasses. Soil type was not studied at this site.

**Red Hills**

The Red Hills site was 30km northeast of Paradise Valley, Nevada. The site was located near an alluvial fan remnant, and the surrounding terrain was a mixture of alluvial fans and low hills. The Red Hills site elevation was 1725m above sea level. Biocrusts were likely common at the site before the 2018 wildfire. Surface soils were loams while the subsurface soils were clay loams.

**Twin Valley**

The Twin Valley site was 28km south of the Idaho border in Nevada. The site terrain was a plateau with rolling hills and low-sloped drainage basins. The elevation at this site was 1668m above sea level. Well-developed biocrusts were likely common at this site before the Martin wildfire in 2018. Surface and subsurface soil textures were silt loam.

**Supplementary Figures**

**Figure S1.** Image of CPER collection site feature an MWAC sampler identical to samplers found at all sites used with permission from the National Wind Erosion Research Network website (Van Zee, n.d.).

**Figure S2.** Alpha rarefaction plot of samples colored by site.
